# Innovative applications and future trends of multiparametric PET in the assessment of immunotherapy efficacy

**DOI:** 10.3389/fonc.2024.1530507

**Published:** 2025-01-20

**Authors:** Tingting Qiao, Zhaoping Cheng, Yanhua Duan

**Affiliations:** ^1^ Department of Nuclear Medicine, The First Affiliated Hospital of Shandong First Medical University, Jinan, China; ^2^ Graduate School, Shandong First Medical University, Jinan, China

**Keywords:** multiparametric positron emission tomography, immunotherapy, tumor microenvironment, prognostic prediction, multi-omics integration

## Abstract

**Background:**

The integration of multiparametric PET (Positron Emission Tomography.) imaging and multi-omics data has demonstrated significant clinical potential in predicting the efficacy of cancer immunotherapies. However, the specific predictive power and underlying mechanisms remain unclear.

**Objective:**

This review systematically evaluates the application of multiparametric PET imaging metrics (e.g., SUVmax [Maximum Standardized Uptake Value], MTV [Metabolic Tumor Volume], and TLG [Total Lesion Glycolysis]) in predicting the efficacy of immunotherapies, including PD-1/PD-L1 inhibitors and CAR-T therapy, and explores their potential role in improving predictive accuracy when integrated with multi-omics data.

**Methods:**

A systematic search of PubMed, Embase, and Web of Science databases identified studies evaluating the efficacy of immunotherapy using longitudinal PET/CT data and RECIST or iRECIST criteria. Only original prospective or retrospective studies were included for analysis. Review articles and meta-analyses were consulted for additional references but excluded from quantitative analysis. Studies lacking standardized efficacy evaluations were excluded to ensure data integrity and quality.

**Results:**

Multiparametric PET imaging metrics exhibited high predictive capability for efficacy across various immunotherapies. Metabolic parameters such as SUVmax, MTV, and TLG were significantly correlated with treatment response rates, progression-free survival (PFS), and overall survival (OS). The integration of multi-omics data (including genomics and proteomics) with PET imaging enhanced the sensitivity and accuracy of efficacy prediction. Through integrated analysis, PET metabolic parameters demonstrated potential in predicting immune therapy response patterns, such as pseudo-progression and hyper-progression.

**Conclusion:**

The integration of multiparametric PET imaging and multi-omics data holds broad potential for predicting the efficacy of immunotherapies and may support the development of personalized treatment strategies. Future validation using large-scale, multicenter datasets is needed to further advance precision medicine in cancer immunotherapy.

## 1 Introduction

The introduction of immune checkpoint inhibitors (ICIs) has revolutionized cancer treatment, significantly improving outcomes in patients with advanced-stage cancer by enhancing immune surveillance to combat tumor growth. These therapies have proven effective in prolonging progression-free survival (PFS) and overall survival (OS), but evaluating their efficacy presents unique challenges. Traditional imaging techniques like Computed Tomography (CT) and Magnetic Resonance Imaging (MRI) typically measure changes in tumor size, which may not accurately reflect the therapeutic response in the context of immunotherapy. This is particularly true for immune-related phenomena such as pseudoprogression and hyperprogression, where tumor volume may not change immediately or may increase before a subsequent reduction, complicating response evaluation.

Multiparametric PET/CT has emerged as a powerful tool in assessing treatment efficacy by providing functional insights into tumor metabolism and immune responses within the tumor microenvironment (TME). Unlike conventional imaging, PET/CT can capture early metabolic alterations and immune cell infiltration, offering a more comprehensive picture of treatment effects before morphological changes are visible (1, 2). Key semi-quantitative parameters like SUVmax, MTV, and TLG reflect shifts in metabolic activity and can identify early signs of treatment response, while PET-derived markers such as PD-L1 expression and CD8-positive T cell infiltration offer further insights into the immunological dynamics of the TME (3–5).

This review examines the role of multiparametric PET/CT in evaluating immunotherapy outcomes, with a focus on PET-derived metabolic parameters and immune responses to inform clinical decision-making (**Table 1**). It also discusses the limitations of traditional imaging in detecting immune-related changes and reviews the RECIST and iRECIST criteria for evaluating immunotherapy responses. Concepts such as pseudoprogression and hyperprogression will be discussed within this context, highlighting the potential of PET/CT to detect these atypical patterns of tumor response, thus offering a more accurate early assessment of immunotherapy efficacy.

**Table 1:** Summary of clinical data related to immunotherapy.

Types of Immunotherapy	Efficacy Indicators	PET: SUVmax	PET: TLG
PD-1/PD-L1 Inhibitors	OS、PFS	An increase in SUVmax is associated with therapeutic efficacy.	The change in TLG is associated with immune response assessment.
CAR-T Cell Therapy	Complete Remission (CR)、Partial Remission (PR)	SUVmax cannot directly predict therapeutic outcomes.	No significant change in TLG was observed.
Immune Checkpoint Inhibitors	PFS Duration of Response (DOR)	SUVmax is correlated with the type of response.	A decrease in TLG is associated with tumor progression.
Cytokine Therapy	Immune Cell Infiltration Cytokine Levels	Elevated SUVmax is associated with enhanced immune response.	An increase in TLG suggests active immune response.
Vaccine Therapy	Antigen-Specific Immune Response	There is a correlation between SUVmax and therapeutic efficacy.	The predictive value of TLG for therapeutic efficacy is limited.

## 2 Materials and methods

### 2.1 Literature search strategy and inclusion criteria

A comprehensive literature search was conducted across PubMed, Embase, Web of Science, and the Cochrane Library for studies published from 1925 to April 2024. The search included terms such as “multiparametric PET,” “SUVmax,” “MTV,” “TLG,” “immunotherapy,” “CAR-T therapy,” “RECIST,” and “iRECIST.” Eligible studies involved adult cancer patients receiving immunotherapy with longitudinal [18F] FDG PET/CT scans, and used RECIST or iRECIST criteria for tumor evaluation and response assessment.

#### 2.1.1 Inclusion criteria

Eligible studies involved adult cancer patients receiving immunotherapy, with longitudinal PET/CT scans performed. Studies were required to report both morphological and metabolic response data, evaluated using RECIST or iRECIST criteria, and to include clinical outcomes such as PFS or OS.

#### 2.1.2 Exclusion criteria

Studies were excluded if they did not use standardized RECIST or iRECIST criteria, had inadequate imaging quality or incomplete patient data, were non-clinical (e.g., animal or *in vitro* studies), or had small sample sizes (n < 30) or insufficient follow-up.

### 2.2 Data extraction and quality assessment

Key data extracted included patient baseline characteristics, PET imaging metrics (e.g., SUVmax, MTV, TLG), multi-omics data, efficacy evaluation standards (RECIST or iRECIST), and survival outcomes (e.g., PFS, OS). Two independent reviewers conducted data extraction and quality assessment using the Newcastle-Ottawa Scale (NOS) to ensure methodological rigor and reliability.

### 2.3 Limitations and potential biases

While RECIST and iRECIST are essential for assessing tumor progression, several limitations and biases should be acknowledged. Variations in the quality of [18F]FDG PET/CT imaging—such as differences in scan resolution, imaging protocols, and patient preparation—can affect the consistency of results. Methodological discrepancies, including variations in lesion size measurement and timing of evaluations, may also hinder result comparability. Additionally, selection bias is a concern as studies often focus on specific cancer types or treatment regimens, potentially overrepresenting immunotherapy responders. Lastly, distinguishing pseudoprogression from true progression remains challenging, as immune-related changes in the tumor microenvironment can lead to false positives, misclassifying immune responses as tumor progression.

## 3 The role of PET/CT imaging in tumor immunotherapy

### 3.1 The role of multiparametric PET in assessment standards

Multiparametric PET imaging, including key metrics such as SUVmax, MTV, and TLG, plays a crucial role in assessing tumor metabolism and immune responses within the TME during immunotherapy. While SUVmax reflects the peak metabolic activity, it does not capture intratumoral heterogeneity, whereas MTV and TLG provide a more comprehensive view of the tumor’s metabolic burden and are recognized as significant prognostic factors in lung cancer immunotherapy. These metrics help in monitoring immune-related changes, such as pseudo-progression and hyper-progression, thus offering more accurate treatment response assessments.

**Figure 1:**
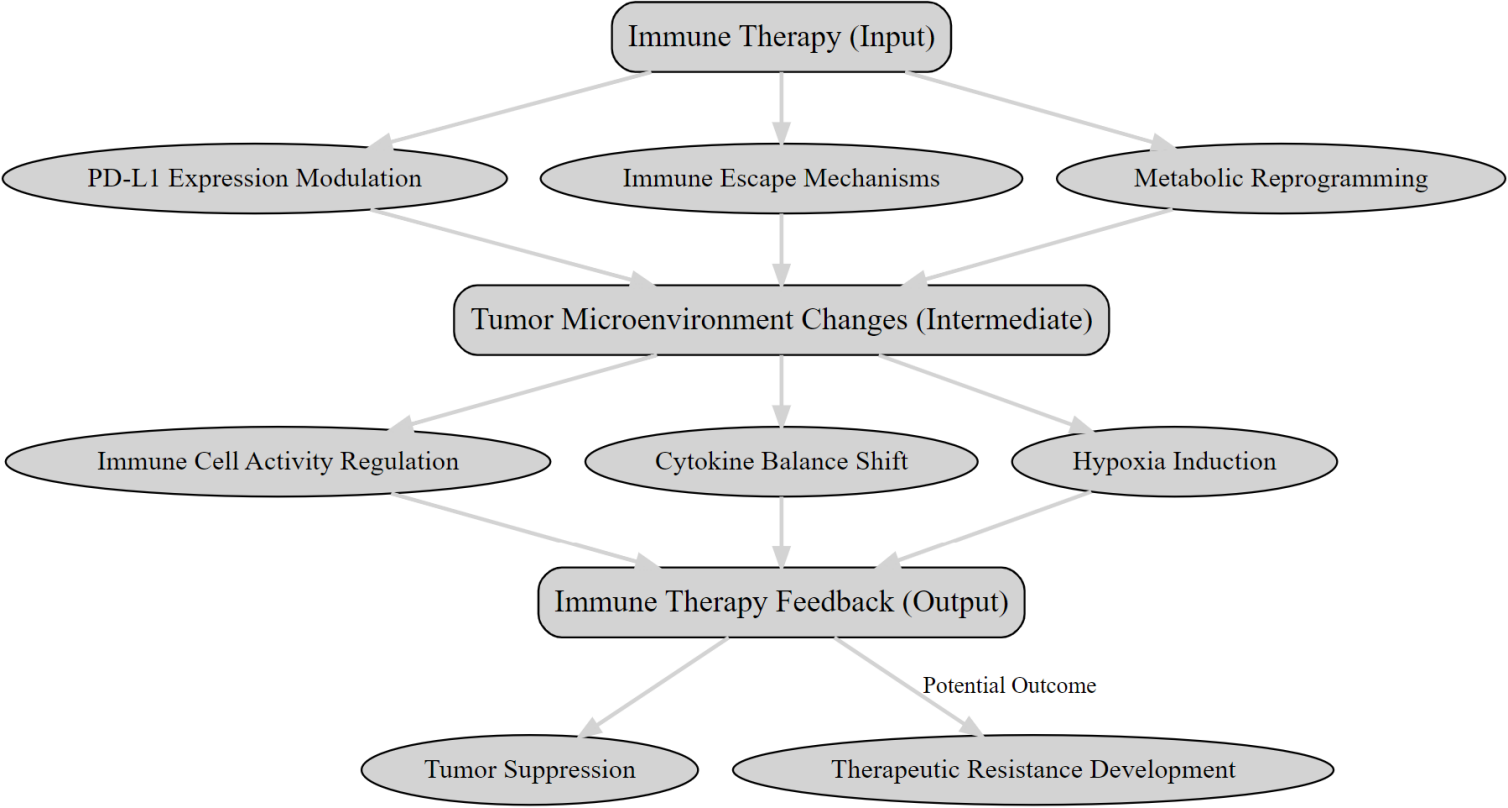
The feedback mechanisms between immunotherapy and the tumor microenvironment.

The TME is central to the efficacy of immunotherapies like PD-1/PD-L1 inhibitors and CAR-T cell therapies, influencing immune activation and tumor progression through complex feedback mechanisms (as illustrated in **Figure 1**). These mechanisms affect treatment outcomes, and integrating PET with molecular and immunological data can optimize therapeutic strategies and improve patient outcomes.

In lung cancer immunotherapy, whole-body MTV has been shown to predict prognosis, with higher MTV values correlating with more aggressive tumors and greater likelihood of disease progression. For example, baseline total MTV on PET is a predictor of overall survival (AUC=0.64), and mid-treatment MTV is a stronger prognostic marker for survival and progression-free survival (AUCs of 0.83 and 0.82, respectively). These findings highlight the value of residual TMTV after 6–8 weeks of immune checkpoint inhibitor therapy as an independent prognostic indicator (6).

Studies also show that PET/CT imaging parameters are associated with immune therapy outcomes. For instance, Seban et al. (2020) found that blood inflammatory markers, such as the Derived Neutrophil-to-Lymphocyte Ratio (dNLR), Platelet-to-Lymphocyte Ratio (PLR), and C-reactive Protein (CRP), correlated with PFS and OS in Non-Small Cell Lung Cancer (NSCLC) patients undergoing immunotherapy or chemotherapy. Specifically, high dNLR, SII, and SLR were independent prognostic factors for PFS and OS in the immunotherapy cohort (7). Furthermore, Monaco et al. (2020) found that baseline MTV is an independent predictor of response to ICIs. Patients achieving disease control (complete response, partial response, or stable disease) had significantly lower median MTV values compared to those with progressive disease (77 vs. 160.2, p = 0.039). Additionally, lower MTV and TLG values were associated with improved OS (p = 0.03 and 0.05, respectively) (8). Similarly, Ito et al. (2020) demonstrated that melanoma patients with MTV values above the median had significantly shorter OS compared to those with values below the median. The median OS for all patients was 14.7 months, with MTV serving as a strong independent prognostic factor (p = 0.001). Patients with MTV above the median had a median survival of 10.8 months, while those with MTV below the median had a median survival of 26.0 months (9).

### 3.2 Development and application of novel PET radiotracers

The growth and survival of cancer cells are driven by their unique metabolic features, particularly the Warburg effect, where cells preferentially undergo aerobic glycolysis even in the presence of oxygen (10). This metabolic shift results in increased glucose uptake and lactate production, a hallmark that can be detected using [18F]FDG PET imaging. While [18F] FDG is widely used to assess tumor metabolism, it has limitations in evaluating immunotherapy efficacy, particularly in distinguishing between pseudoprogression and true progression. To address these challenges, there is growing interest in developing novel PET radiotracers that target immune-related biomarkers, which can provide more specific and dynamic insights into tumor immune responses and progression during immunotherapy.

Recent advancements have led to the development of PET radiotracers targeting key immune markers such as PD-L1, CD8+ T cells, and regulatory T cells (Tregs). These tracers enable non-invasive visualization of immune activity within the TME, providing valuable information on immune cell infiltration, activation, and response to therapy (11).

An overview of these tracers is provided in **Table 2**.

**Table 2:** Overview of the application of PET tracers in immunotherapy.

PET Tracers	Indicated Therapy	Mechanism of Action	Potential Applications	Preclinical Research Progress	Clinical Trial Progress
[18F] FDG	Cancer Immunotherapy	Metabolically Active Tumor Cells	Monitoring Tumor Metabolic Activity	Effective Monitoring of Tumor Metabolism	Clinical Research Phase: Evaluation of Tumor Metabolic Monitoring
[18F] FLT	Cancer Immunotherapy	Cell Proliferation	Monitoring Tumor Cell Proliferation	Effective Monitoring of Proliferating Tumor Cells	In Clinical Trials: Used for Proliferating Tumors
Anti-CD25 Antibody-Tagged Tracers	Immune Cell Monitoring	Immune Cell Activity	Monitoring Treg Cell Activity	Successful Treg Cell Labeling	Preclinical Research: Evaluation of Treg Cell Monitoring
[11C] choline	Cancer Immunotherapy	Cell Membrane Synthesis	Monitoring Tumor Cell Membrane Synthesis	Used for Monitoring Tumor Cell Membrane Synthesis	Currently in Early Clinical Trial Phase
[18F] FMISO	Cancer Immunotherapy	Hypoxic Microenvironment	Monitoring Tumor Hypoxic Regions	Successful Identification of Hypoxic Microenvironment	Ongoing Clinical Trials for Hypoxic Labeling

#### 3.2.1 PD-L1-targeted PET radiotracers

PD-L1 expression is a critical factor in determining response to ICIs. PET tracers targeting PD-L1, such as [68Ga]-NOTA-WL12, have demonstrated promising results in predicting immunotherapy efficacy. For instance, Zhou et al. (2021) conducted the first human study to evaluate [68Ga]-NOTA-WL12 as a non-invasive PET radiotracer for *in vivo* detection of tumor PD-L1 expression. A strong positive correlation was observed between tumor uptake (SUVpeak) and PD-L1 immunohistochemistry (r = 0.9349; P = 0.002), which suggesting that PD-L1 PET could help predict the response to pembrolizumab in combination with chemotherapy. However, a limitation of this study was the reliance on immunohistochemistry from a single lesion, without addressing expression variability across multiple lesions (12).Similarly, Liu et al. (2022) demonstrated that [68Ga] Ga-NOTA-Nb109 effectively detected PD-L1 expression in NSCLC xenografts, supporting its potential as a tool for patient selection in immunotherapy. These findings highlight how PD-L1-targeted PET can guide personalized treatment by identifying patients who are likely to benefit from PD-L1-targeted therapies (13).

#### 3.2.2 CD3 and CD8+ T cell-targeted PET radiotracers

CD3 and CD8+ T cells play a central role in the immune response against tumors, making them key targets for monitoring the effects of immunotherapy. In a study by Benjamin M. Larimer et al. (2020), CD3-targeted PET radiotracers demonstrated a strong correlation between high radiotracer uptake and tumor volume reduction in a mouse model undergoing anti-CTLA-4 therapy. This suggests that CD3 PET imaging could be a promising tool for evaluating the therapeutic efficacy of immunotherapy (14). Michael D. Farwell et al. (2022) conducted a phase 1 human PET imaging study using an anti-CD8 radiolabeled mini-antibody, [89Zr] Df-IAB22M2C. The study successfully visualized the distribution of CD8+ T cells in both tumors and reference tissues of patients with metastatic solid tumors, demonstrating the potential of this approach for predicting early responses to immunotherapy (15). This work provides critical technical support for the quantitative monitoring of CD8+ T cells and facilitates the early evaluation of immune therapy responses during treatment.

#### 3.2.3 Emerging immune-related PET radiotracers

Tregs are known to suppress immune responses and contribute to immune evasion by tumors. Therefore, imaging the density of Tregs within the TME can provide crucial information on the tumor’s ability to evade immune surveillance. Novel PET radiotracers targeting Tregs, such as those using antibodies against specific Treg markers, are being developed to assess immune suppression in tumors. For instance, Elevated Treg levels are associated with metastatic spread to tumor-draining lymph nodes (TDLNs), underscoring the importance of Treg-targeted PET radiotracers for assessing immunotherapy efficacy (16).

TAMs, which are key contributors to immune suppression and tumor progression in the tumor microenvironment, represent a promising target for monitoring the effectiveness of immune-based therapies. Lee et al. (2021) further confirmed that PET radiotracers labeled with anti-CD25 antibodies bind specifically to Tregs and enable successful imaging in murine models. The interleukin-2 receptor α chain (IL-2Rα; CD25) is a promising target for immune therapy and radioimmunotherapy in lymphomas. Immuno-PET can aid in visualizing CD25 expression *in vivo*. Biodistribution studies demonstrated high tumor uptake of 89Zr-CD25 IgG (8.7 ± 0.9%ID/g), surpassing both blood (5.2 ± 1.6%ID/g) and other organ uptakes (0.7 to 3.5%ID/g) (17). Several PET radiotracers designed to label M1 and M2 macrophages are currently undergoing preclinical evaluation (18). The targeting T cell metabolic activity, including [18F]FDG or ^18^F-fluoro-thymidine (FLT), holds significant potential for assessing early responses to immunotherapy (19).

### 3.3 PET imaging in the evaluation of pseudo-progression and true progression during immunotherapy

Traditional tumor response criteria, such as RECIST, are limited in distinguishing pseudo-progression from true progression in cancer patients undergoing immunotherapy. Pseudo-progression refers to temporary tumor growth due to immune cell infiltration and inflammation, which does not reflect true tumor progression (20). To address this, newer criteria like iRECIST and irRC have been developed to better differentiate these phenomena, incorporating confirmatory imaging and a more nuanced approach to the appearance of new lesions. A retrospective study by Masatoyo Nakajo et al. evaluated the predictive value of both the European Organization for Research and Treatment of Cancer (EORTC) and PERCIST criteria in predicting PFS in patients with advanced or metastatic gastric cancer treated with nivolumab (21). PET/CT, particularly [18F] FDG PET, is increasingly used to evaluate metabolic responses during immunotherapy, providing an early indication of treatment effects and helping differentiate pseudoprogression from true progression. Studies show that FDG-PET/CT can predict long-term prognosis, detect secondary progression, and identify immune-related adverse events (irAEs), which may precede clinical symptoms (22).

#### 3.3.1 High progression disease (HPD) in NSCLC

In NSCLC, HPD is associated with poorer outcomes and higher mortality, and it remains a significant challenge in immunotherapy. HPD, occurring in about 9% of advanced cancer patients and 29% of head and neck cancer patients treated with PD-1/PD-L1 inhibitors, may result from abnormal immune responses or immune dysregulation in the tumor microenvironment (23, 24). Identifying HPD requires a multidimensional approach, combining tumor growth patterns, biomarker profiles, and the patient’s immune status, with PET imaging and ctDNA analysis providing valuable predictive data for early identification.

#### 3.3.2 Distinguishing pseudo-progression from hyper-progression in lung cancer: insights from SUVmax trends, EGFR mutations, and PD-L1 expression

**Figure 2:**
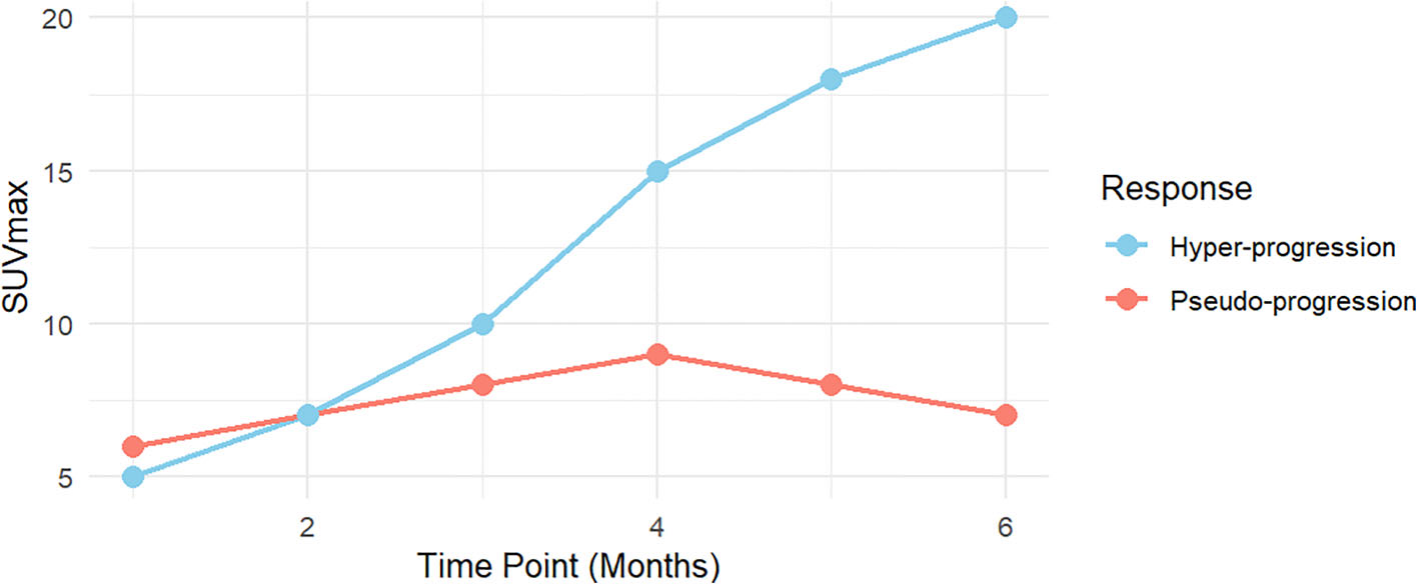
The differences in the trend of SUVmax variations between patients with pseudo-progression and hyper-progression.

Based on the findings regarding pseudo-progression and hyper-progression, this review first explores the differences in the trends of SUVmax variations between patients with pseudo-progression and hyper-progression (**Figure 2**).

**Figure 3:**
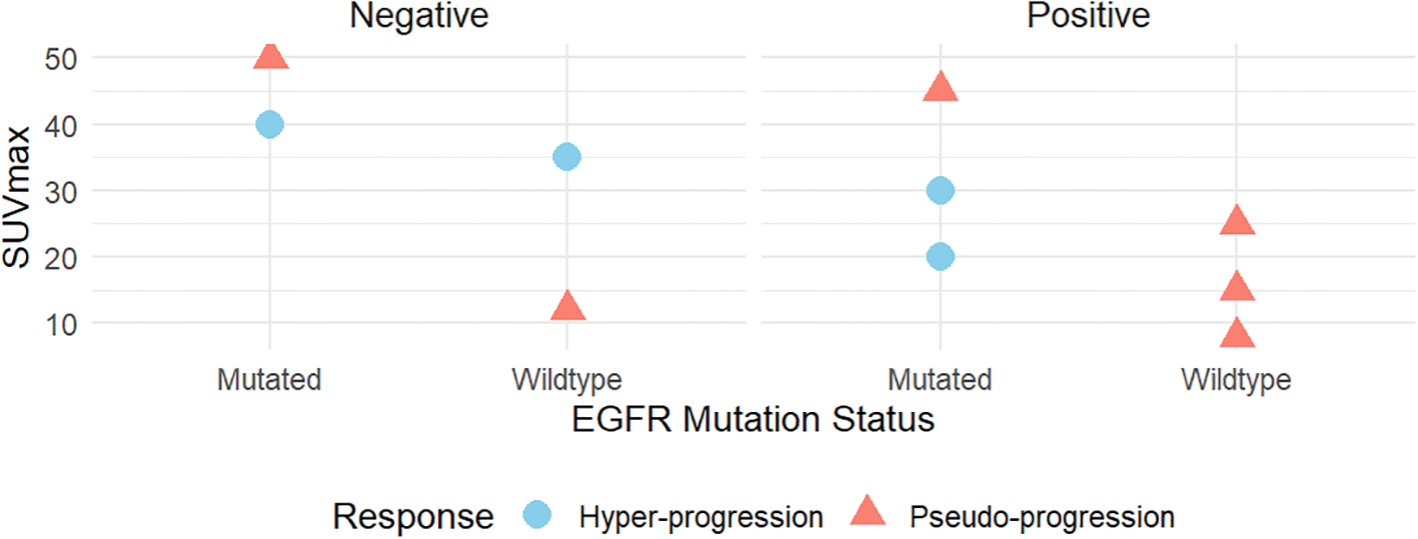
The correlations between SUVmax and immune response type, genetic mutations (EGFR), and PD-L1 expression.

Furthermore, we delve deeper into the relationships between SUVmax, immune response types, EGFR mutation status, and PD-L1 expression, comparing the differences between pseudo-progression and hyper-progression patients (**Figure 3**). High SUVmax values are frequently observed in EGFR-mutated cases, supporting the hypothesis that PD-L1 expression promotes an inflammatory tumor microenvironment, enhancing glucose metabolism and FDG uptake. Lower SUVmax values are typically seen, reflecting reduced immune-related metabolic activity.

##### 3.3.2.1 SUVmax trends in pseudo-progression and hyper-progression

SUVmax trends during immunotherapy typically follow two distinct patterns: pseudo-progression and hyper-progression. In pseudo-progression, the early phase (0–2 months) is marked by a mild increase in SUVmax, primarily due to transient immune activation and inflammation. This mimics tumor progression but does not reflect true tumor growth. During the plateau phase (2–4 months), SUVmax stabilizes or slightly declines, indicating resolution of immune-mediated inflammation without evidence of actual tumor progression. In the late phase (4–6 months), a significant decline in SUVmax suggests an effective therapeutic response, reflecting reduced metabolic activity and immune-mediated tumor control.

In contrast, hyper-progression is distinguished by a rapid and sustained increase in SUVmax over the 0–6 month period, indicating unchecked tumor metabolic activity and aggressive growth. Unlike pseudo-progression, hyper-progression lacks a plateau phase, with the continuous rise in SUVmax reflecting tumor-driven metabolic processes and accelerated proliferation rather than immune-mediated inflammation.

##### 3.3.2.2 Biological mechanisms and clinical implications

The distinct SUVmax trends in pseudo-progression and hyper-progression highlight their biological differences with important clinical implications. In pseudo-progression, early SUVmax increases reflect immune cell infiltration and inflammation induced by ICIs. This transient activation, captured by FDG-PET, stabilizes or declines over time, signifying immune-mediated tumor suppression by T cells and cytokines.

In hyper-progression, SUVmax rises continuously, reflecting aggressive tumor proliferation driven by oncogenic alterations such as EGFR mutations, MDM2 amplification, or TP53 mutations. FDG-PET imaging captures sustained metabolic hyperactivity, independent of immune involvement. These divergent patterns emphasize the necessity of longitudinal SUVmax monitoring to differentiate between immune activation and true tumor progression, enabling precise therapeutic decision-making.

##### 3.3.2.3 Role of EGFR mutations, SUVmax and PD-L1 expression

Elevated SUVmax in pseudo-progression aligns with findings that immune-related metabolic activity can mimic tumor progression. Conversely, moderate SUVmax in hyper-progression more reliably reflects true tumor proliferation, distinguishing it from pseudo-progression. In pseudo-progression, EGFR mutations may drive heightened immune responses, contributing to higher SUVmax values due to increased immune cell recruitment. In hyper-progression, EGFR mutations are likely oncogenic drivers that accelerate tumor growth, independent of immune activity. PD-L1-positive tumors amplify immune responses, increasing metabolic activity detectable on FDG-PET (higher SUVmax), while PD-L1-negative tumors exhibit lower SUVmax levels, correlating with reduced immune-related metabolic activity.

##### 3.3.2.4 Clinical implications for treatment strategies

Integrating SUVmax, EGFR mutation status, and PD-L1 expression provides a comprehensive framework for distinguishing pseudo-progression from hyper-progression in lung cancer. Elevated SUVmax with EGFR mutations and PD-L1 positivity suggests immune-related metabolic activity, where continued immunotherapy may be beneficial despite radiological progression. In contrast, a moderate SUVmax combined with tumor progression markers is indicative of actual tumor growth, thereby necessitating the consideration of alternative therapeutic strategies. This approach is crucial for refining immunotherapy, and large-scale clinical trials are needed to validate and optimize this diagnostic framework.

## 4 Multiparametric PET and radiomics

In recent years, the rapid development of artificial intelligence (AI) and radiomics technologies has substantially enhanced the application of PET in the quantification of tumor heterogeneity and the prediction of treatment responses. Radiomics, an emerging imaging technology, involves the extraction and quantification of high-dimensional features from medical images, offering a powerful tool for elucidating the histological and molecular characteristics of tumors. These features not only enable the assessment of tumor morphology and metabolic activity but also provide insights into the underlying biological heterogeneity, which may not be visible through conventional imaging.

Radiomic features (RF) are primarily categorized into first-order statistical features, such as SUV, MTV, and TLG, as well as higher-provide statistical features, including texture analysis. First-order features offer quantitative measurements of tumor metabolic activity and spatial distribution, while higher-order features, such as texture analysis, capture complex patterns that describe the tumor's internal structure and heterogeneity, helping to assess its spatial relationships and uniformity. Numerous studies have demonstrated that these radiomic features correlate with tumor histological subtypes, genetic mutations (EGFR, KRAS mutations), and immune biomarkers (PD-L1 expression) (25). For example, MTV and TLG, as imaging-derived quantitative metrics, have been shown to be prognostic indicators for clinical outcomes in various cancers, including NSCLC, and can effectively predict patients’ responses to immunotherapy and targeted therapies. Iravani et al. (2020) found that FDG PET-based texture features could effectively distinguish between EGFR mutations in exons 19 and 21, with an AUC of 0.86, sensitivity of 0.84, specificity of 0.73, and accuracy of 0.78 (26–28).

While radiomics provides detailed quantitative data about tumor characteristics, the integration of AI takes this analysis a step further, enabling automated interpretation of complex patterns in imaging data. AI algorithms are capable of automatically extracting texture features from PET scans and classifying tumor subtypes, such as distinguishing between adenocarcinoma and squamous cell carcinoma. Furthermore, AI facilitates the analysis of complex relationships between imaging features, genetic mutations, and immune-related biomarkers, thereby assisting clinicians in identifying potential driver mutations and optimizing patient stratification and personalized treatment strategies (29). The convergence of AI and radiomics is not only advancing tumor classification but also holds significant promise in the context of immunotherapy. For instance, AI-enhanced multiparametric PET analysis has shown potential in predicting the long-term efficacy of immunotherapy, identifying patients most likely to benefit from treatment, and optimizing individualized treatment regimens (30).

It is important to note that multimodal radiomics research is expanding the clinical utility of PET imaging. For example, Zhou et al. (2022) developed the Deep Radiomics Bevacizumab Efficacy Predicting Model (DERBY), which, after incorporating histopathological features, demonstrated robust accuracy for predicting tumor response in an external validation cohort (AUC 0.83, 95% CI [0.75-0.92], sensitivity 80.4%, specificity 76.8%). DERBY also showed prognostic value, with responders exhibiting significantly longer progression-free survival (9.6 vs 6.3 months, p = 0.002) and overall survival (27.6 vs 18.5 months, p = 0.010) compared to non-responders (31, 32). This study highlights the significant potential of combining multiparametric PET with AI and radiomics, which not only enhances tumor assessment accuracy but also plays a critical role in precision medicine. With continued advancements in data acquisition, computational capabilities, and analytical algorithms, these multimodal approaches are expected to increasingly influence the management of a wide range of tumor types and therapeutic strategies in the near future.

## 5 Integration of multiparametric PET imaging and multi-omics data

### 5.1 Integration of multi-omics and PET imaging for precision oncology

The integration of multiparametric PET imaging with multi-omics data, including genomics, proteomics, and metabolomics, has transformed cancer research by offering a more holistic understanding of tumor biology. This convergence of imaging and molecular data provides a comprehensive view of tumor characteristics, enabling precise treatment planning and personalized therapeutic strategies.

Genomic data, such as driver mutations (e.g., EGFR and ALK in non-small cell lung cancer), plays a pivotal role in guiding targeted therapies, influencing patient responses and shaping treatment decisions (33). Proteomics, which investigates protein expression and interactions within both tumor cells and their microenvironment, provides valuable insights into immune evasion, tumor progression, and therapeutic resistance. This understanding is crucial for predicting responses to immunotherapy and other targeted treatments, emphasizing the importance of the tumor immune microenvironment in clinical decision-making. By combining these omics layers with PET imaging, clinicians can achieve a more accurate and individualized approach to cancer treatment.

### 5.2 Metabolic heterogeneity and machine learning in predicting cancer treatment responses

The integration of PET imaging with multi-omics data, particularly genomics, proteomics, and metabolomics, provides a comprehensive approach to understanding tumor metabolic heterogeneity and predicting therapeutic responses. AI plays a crucial role in leveraging the synergies between PET imaging parameters and molecular data to enhance the precision of treatment planning. Emerging evidence has shown a strong correlation between PET imaging features and specific genomic alterations. For instance, EGFR mutations are frequently associated with increased SUVmax, reflecting higher metabolic activity in certain tumor subtypes. Moreover, MTV and TLG, which quantify overall metabolic activity and heterogeneity, are valuable for predicting responses to immunotherapy (34).

Proteomic and metabolomic data provide the biological context for these imaging parameters, offering deeper insights into how different tumor subtypes respond to treatments. The combination of these multi-omics platforms with immunotherapy data can help identify biomarkers that guide clinical decision-making, allowing for more precise and individualized therapeutic strategies.

Machine learning models are further enhancing the integration of PET imaging and multi-omics data. The MONDRIAN study, for example, demonstrated the potential of machine learning algorithms in predicting early-stage NSCLC patients’ responses to stereotactic body radiation therapy (SBRT). These algorithms also help identify features of the tumor microenvironment, predict responses to immunotherapy, and evaluate mechanisms of immune resistance (35). By refining treatment response predictions, machine learning technologies provide robust support for tailoring individualized treatment plans, improving clinical outcomes in cancer therapy.

### 5.3 Impact of multi-omics integration on clinical outcomes

The integration of multiparametric PET imaging with multi-omics data is increasingly recognized as a promising strategy in clinical oncology. This approach enhances the predictive accuracy of therapeutic responses, particularly for immunotherapy, in cancers such as lung cancer and melanoma. By combining imaging data with genomic, proteomic, and metabolomic information, it offers deeper insights into tumor biology, facilitates the identification of novel biomarkers, and supports earlier diagnosis and precision treatment (36).

In cancers such as lung cancer and melanoma, this integrated approach has demonstrated significant clinical success, improving patient stratification and enabling more targeted and personalized therapies. As these methodologies continue to evolve, clinicians will be better equipped to tailor treatments that maximize treatment efficacy, minimize adverse effects, and ultimately enhance patient outcomes.

## 6 Advances, challenges, and perspectives on the integration of multiparametric PET imaging and multi-omics data in immunotherapy

In recent years, considerable progress has been made in integrating multiparametric PET imaging with multi-omics data in the context of cancer immunotherapy. PET imaging provides critical metabolic and functional insights into tumors, serving as a robust tool for monitoring and predicting therapeutic responses. Furthermore, multi-omics analysis, which includes genomics, proteomics, and metabolomics, enhances the molecular context provided by PET, thereby facilitating the development of personalized treatment strategies. This synergistic integration supports the prediction of immunotherapy efficacy, refinement of patient stratification, and identification of specific biomarkers for precision medicine (37). For instance, SUVmax has been closely associated with tumor mutational burden (TMB), and higher TMB correlates with improved responses to ICIs (38). Additionally, features of the TME, such as immune cell infiltration and immunosuppressive states, can be inferred from PET-derived metabolic characteristics, including elevated FDG uptake, which may serve as an early indicator of immunotherapy response.

### 6.1 Discovery of biomarkers

The integration of PET imaging with multi-omics data has led to the identification of several promising biomarkers associated with immunotherapy outcomes. For example, the combination of genomic data (PD-L1 expression, EGFR mutations) with PET imaging features can help predict which patients are likely to respond favorably to PD-1/PD-L1 inhibitors (39–41). Proteomic analyses have also revealed biomarkers related to immune evasion mechanisms within the TME, allowing for more precise patient stratification. Specific immune evasion-related proteins, such as CTLA-4, TIM-3, and LAG-3, have been shown to correlate with therapeutic efficacy, thereby contributing to improved patient selection and the personalization of treatment regimens.

### 6.2 Application of machine learning models

Machine learning, particularly deep learning, has become instrumental in the integration of multiparametric PET imaging and multi-omics data. Deep learning algorithms can automatically extract complex patterns from imaging data and combine these with genomic, proteomic, and other omics data to predict responses to immunotherapy. For instance, certain deep learning models have been developed to predict immune cell infiltration within the TME based on PET imaging features, thereby providing insight into the potential efficacy of immunotherapy.

### 6.3 Challenges in data integration

Despite the vast potential of integrating multiparametric PET imaging with multi-omics data, several challenges remain in the data integration process. Different data types, including imaging data, genomic data, and proteomic data, exhibit significant variations in their formats, scales, and analysis methodologies. One of the major hurdles is the effective standardization and preprocessing of data, which are essential to ensure compatibility and consistency across heterogeneous data sources. Furthermore, variations in data quality and accuracy, arising from the use of different imaging equipment, technological platforms, and databases, pose significant challenges. Addressing these issues and ensuring the reliability and consistency of data are crucial for the successful clinical integration of multi-omics data.

Although studies have demonstrated the potential of combining PET imaging with multi-omics data, differences in technological methods, analytical tools, and evaluation criteria across studies have led to inconsistencies in data integration and model standardization. This variability not only impairs reproducibility across platforms but also limits the comparability and validation of findings across different research endeavors. Therefore, developing standardized data processing methodologies and protocols for cross-platform data integration, along with rigorous model validation, will be essential for advancing research in this field.

### 6.4 Cost and technical barriers

The acquisition and processing of multiparametric PET imaging and multi-omics data are resource-intensive and demand significant technical expertise. The high cost associated with PET imaging equipment and multi-omics platforms limits their widespread use in resource-constrained clinical settings. Moreover, multi-omics data analysis requires substantial computational resources and sophisticated algorithms, presenting a considerable technical barrier to clinical adoption. Reducing costs, optimizing workflows, and developing more accessible, efficient data processing tools will be critical for enabling the broader clinical application of these technologies.

### 6.5 Future directions

Looking forward, advancements in high-resolution PET imaging and improved multi-omics data processing techniques hold the potential to enhance data precision and integration efficiency. Future research efforts should focus on the multidimensional integration of genomic, proteomic, and metabolic biomarkers to identify biological features closely associated with immunotherapy outcomes, thereby enabling the development of more precise therapeutic strategies.

## 7 Conclusion

In conclusion, the combination of multiparametric PET imaging with immunotherapy, alongside the integration of multi-omics data, represents a promising frontier in precision cancer treatment. PET imaging effectively quantifies tumor metabolic activity and heterogeneity, showcasing its potential in assessing immunotherapy responses. With the advent of artificial intelligence, PET imaging has transcended traditional analysis methods, allowing for precise predictions of immunotherapy efficacy and fostering personalized treatment approaches. Additionally, the integration of PET with genomic data deepens our understanding of the tumor immune microenvironment, aids in the discovery of novel biomarkers, and offers non-invasive, dynamic assessments of treatment responses, thus informing the optimization of immunotherapy. Despite ongoing challenges related to standardization and interdisciplinary collaboration, the integration of multiparametric PET imaging with multi-omics data, driven by machine learning and artificial intelligence, is poised to play a transformative role in the future of precision medicine.
